# Variable Carbon Source Utilization, Stress Resistance, and Virulence Profiles Among *Listeria monocytogenes* Strains Responsible for Listeriosis Outbreaks in Switzerland

**DOI:** 10.3389/fmicb.2019.00957

**Published:** 2019-05-03

**Authors:** Francis Muchaamba, Athmanya K. Eshwar, Marc J. A. Stevens, Ueli von Ah, Taurai Tasara

**Affiliations:** ^1^Institute for Food Safety and Hygiene, Vetsuisse Faculty, University of Zürich, Zurich, Switzerland; ^2^Agroscope, Bern, Switzerland

**Keywords:** *Listeria monocytogenes*, phenotype microarray, phenotype, genome, carbon source, stress, virulence

## Abstract

A combination of phenotype microarrays, targeted stress resistance and virulence assays and comparative genome analysis was used to compare a set of *Listeria monocytogenes* strains including those involved in previous Swiss foodborne listeriosis outbreaks. Despite being highly syntenic in gene content these strains showed significant phenotypic variation in utilization of different carbon (C)-sources as well as in resistance of osmotic and pH stress conditions that are relevant to host and food associated environments. An outbreak strain from the 2005 Swiss Tomme cheese listeriosis outbreak (Lm3163) showed the highest versatility in C-sources utilized whereas the strain responsible for the 1983 to 1987 Vacherin Montd’or cheese listeriosis outbreak (LL195) showed the highest tolerance to both osmotic and pH stress conditions among the examined strains. Inclusion of L-norvaline led to enhanced resistance of acidic stress in all the examined strains and there were strain–strain-specific differences observed in the ability of other amino acids and urea to enhance acid stress resistance in *L. monocytogenes*. A strain dependent inhibition pattern was also observed upon inclusion of β-phenylethylamine under alkaline stress conditions. In targeted phenotypic analysis the strain-specific differences in salt stress tolerance uncovered in phenotypic microarrays were corroborated and variations in host cell invasion and virulence among the examined strains were also revealed. Outbreak associated strains representing lineage I serotype 4b showed superior pathogenicity in a zebrafish infection model whilst Lm3163 a lineage II serotype 1/2a outbreak strain demonstrated the highest cellular invasion capacity amongst the tested strains. A genome wide sequence comparison of the strains only revealed few genetic differences between the strains suggesting that variations in gene regulation and expression are largely responsible for the phenotypic differences revealed among the examined strains. Our results have generated data that provides a potential basis for the future design of improved *Listeria* specific media to enhance routine detection and isolation of this pathogen as well as provide knowledge for developing novel methods for its control in food.

## Introduction

*Listeria monocytogenes* is an important foodborne pathogen that accounts for serious public health problems and food safety challenges since it causes severe clinical illnesses and high mortality in vulnerable human populations (European Food Safety Authority [EFSA], 2017; Centers for Disease Control [CDC], 2018; Radoshevich and Cossart, 2018). In high risk groups including young and old people as well as pregnant women infections can manifest as life-threatening meningitis, septicemia, and feto-maternal complications (European Food Safety Authority [EFSA], 2017; Radoshevich and Cossart, 2018). *L. monocytogenes* is a genetically diverse bacterial species that is subdivided into thirteen serotypes, four main evolutionary genetic lineages and numerous MLST clones (Orsi et al., 2011; Haase et al., 2014; Maury et al., 2016). All *L. monocytogenes* strains are presumed equally virulent although the molecular epidemiological evidence gathered to date suggests otherwise. A variable distribution of *L. monocytogenes* genotypes and serological subtypes in food products and processing environments as well as among human and animal clinical listeriosis cases has been reported (Liu, 2008; Orsi et al., 2011; Maury et al., 2016; Moura et al., 2016).

Natural stress resistance and virulence capacity both contribute to current challenges posed by *L. monocytogenes* to public health and food safety (Gandhi and Chikindas, 2007; Toledo-Arana et al., 2009; Allerberger and Wagner, 2010; Soni et al., 2011; Kocaman and Sarımehmetoğlu, 2016; European Food Safety Authority [EFSA], 2017; Radoshevich and Cossart, 2018). In addition, the ability of this pathogen to efficiently exploit various nutrient sources in food and infected host associated environments is crucial for its survival and growth in such environments (Deutscher et al., 2014). *L. monocytogenes* has evolved various systems necessary for nutrient acquisition and utilization, stress adaption and virulence responses that allow for stress survival and transmission along the food chain as well as subsequent host infection and pathogenicity processes (Nadon et al., 2002; Desvaux and Hebraud, 2006; Wilson et al., 2006; Toledo-Arana et al., 2009; Allerberger and Wagner, 2010; Soni et al., 2011; Deutscher et al., 2014; Kocaman and Sarımehmetoğlu, 2016; Radoshevich and Cossart, 2018). Such evolution has generated natural populations of this pathogen that are phenotypically heterogeneous. Nutrient utilization and stress resistance mechanisms documented in this bacterium include numerous enzymes, transporter systems and gene expression regulating proteins (Schmid et al., 2009; Toledo-Arana et al., 2009; Loepfe et al., 2010; Scharer et al., 2013; NicAogáin and O’Byrne, 2016; Chen et al., 2017). The virulence strategies employed in this bacterium involves various proteins that are primarily regulated through the transcription regulator PrfA (positive regulatory factor A), (de las Heras et al., 2011; Radoshevich and Cossart, 2018). PrfA activity is controlled by several environmental signals at the transcriptional and post-transcriptional level, which include temperature and the presence or absence of efficiently metabolized C-sources transported via the phosphoenolpyruvate (PEP): carbohydrate phosphotransferase system (PTS) (Johansson et al., 2002; Joseph et al., 2008). The uptake and metabolism of these carbohydrates leads to strong inhibition of PrfA activity without affecting *prfA* gene expression (Joseph et al., 2008). PrfA expression is also controlled through stress response regulatory proteins such as Sigma B and Csps (Ollinger et al., 2009; Scharer et al., 2013; Eshwar et al., 2017). Therefore, carbon source utilization and stress resistance profiles could be correlated to virulence. As such efforts to determine the global phenome of *L. monocytogenes* strains are important since virulence seems intricately connected to nutrient utilization and stress tolerance.

Several listeriosis outbreaks have been documented in Switzerland to date, which besides causing severe illnesses and claiming lives of several people have also been responsible for significant food safety problems and economic losses to the food industry (Bille, 1990; Bula et al., 1995; Bille et al., 2006; Schmid and Baumgartner, 2012; Hachler et al., 2013; Althaus et al., 2014; Ebner et al., 2015; Stephan et al., 2015; Althaus et al., 2017; Meier et al., 2017). Our current understanding of nutrient exploitation and stress resistance within the host and food-associated environments in outbreak-associated *L. monocytogenes* strains is still limited. A detailed study of metabolism and stress resilience in such strains might, however, provide more clues on the roles played by established and novel physiological and molecular response mechanisms of this bacterium in facilitating colonization, survival and proliferation in food and host associated environments. In this study, a selection of *L. monocytogenes* isolates that includes strains linked to Swiss listeriosis outbreaks and associated food sources were compared with respect to metabolism of different C-sources as well as pH and osmotic stress resistance profiles. A potential association between phenotypic diversity in carbon metabolism and stress resistance with virulence and the genome in such strains was also examined.

## Materials and Methods

### Ethics Statement

This study was carried out in accordance with the principles and recommendations of the “Ordinance on laboratory animal husbandry, the production of genetically modified animals and the methods of animal experimentation; Animal Experimentation Ordinance” (SR 455.163, April 12, 2010), Swiss Federal Food Safety and Veterinary Office (FSVO/BLV). The maximum age reached by the embryos during experimentation was 5 dpf for which no license was required from the cantonal veterinary office in Switzerland, since embryos had not yet reached free feeding stage. Husbandry and breeding of the adult zebrafishes was performed under the supervision of Prof. Stephan Neuhauss, Institute for Molecular Life Sciences, University of Zürich, Zurich, Switzerland. All animal protocols used were in compliance with internationally recognized and with Swiss legal ethical guidelines for the use of fish in biomedical research and experiments were approved by the local authorities (Veterinäramt Zürich Tierhaltungsnummer 150).

### Overview of the Analyzed Strains

A set of strains that includes isolates from past Swiss listeriosis outbreaks as well as some food derived isolates and the *L. monocytogenes* EGDe reference strain was analyzed (Table 1). Outbreak strains included were responsible for previous Swiss Vacherin Montd’or cheese (1983–1987; LL195), Tomme cheese (2005; Lm3163 and Lm3136), ready-to-eat salad (2013–2014; N2306), and meat pâté (2016; N16-0044) listeriosis outbreaks (Bula et al., 1995; Bille et al., 2006; Stephan et al., 2015; Althaus et al., 2017). Two strains randomly isolated from Swiss milk products sampled in 2011 (N14-0435) and 2014 (N11-1515) were included as background strains (Ebner et al., 2015). Strains examined represent different *L. monocytogenes* genetic lineages I and II as well as different MLST clonal complexes and sequence types. LL195, N2306 and N16-0044 are lineage I, serotype 4b strains whist Lm3163 and Lm3136 are lineage II, serotype 1/2a strains. N14-0435 and N11-1515 included as background strains belong to lineage I, serotype 1/2b and lineage II, serotype 1/2a respectively. In addition, *L. monocytogenes* EGDe a lineage II, serotype 1/2a strain was included in the study as a reference strain and *L. innocua* J5051 was included as a negative control in some of the experiments (Guldimann et al., 2015).

**Table 1:** Strains used in this study.

		MLST
Strain ID	Serotype	genotype	Source	Remarks	Reference
EGDe	1/2a	CC9	Rabbits	Reference strain	Glaser et al., 2001
LL195	4b	CC1	Vacherin Montd’or cheese	1983–1987 outbreak	Bille, 1990
Lm3136	1/2a	CC18	Tomme cheese	2005 outbreak	Bille et al., 2006
Lm3163	1/2a	CC26	Tomme cheese	2005 outbreak	Bille et al., 2006
N2306	4b	CC4	Ready-to-eat salads	2013–2014 outbreak	Stephan et al., 2015
N16-0044	4b	CC6	Meat pâté	2016 outbreak	Althaus et al., 2017
N11-1515	1/2a	CC29	Milk isolate	Routine check 2011	Ebner et al., 2015
N14-0435	1/2b	CC3	Milk isolate	Routine check 2014	Ebner et al., 2015

### Bacterial Culture and Growth Conditions

All strains were stored at -80°C in brain heart infusion medium (BHI, Oxoid, United Kingdom) supplemented with 20% glycerol. To prepare inoculum the frozen stock of each strain was streaked out on blood agar or BHI agar plates and incubated overnight at 37°C. Single colonies picked from each plate were then pre-cultured twice in BHI broth (37°C, 150 rpm) for 16 h. Secondary stationary phase cultures generated in this way were subsequently used in experiments unless otherwise stated.

### Phenotype Microarray Analysis

Biolog phenotype microarrays (PMs) were used to compare metabolic and stress tolerance profiles among the strains^1^ (Bochner, 2009). The carbon source (C-source) utilization (PM01 and PM02) as well as osmotic and pH stress (PM09 and PM10) resistance phenotypic profiles were analyzed. PM experiments were done in accordance with standard Biolog Inc. protocols with a few modifications (Fox and Jordan, 2014; see text footnote 1). Bacteria from glycerol stocks stored at -80°C were grown overnight at 37°C on BHI agar. Single colonies of the bacteria grown on BHI agar were sub-cultured on BHI agar plates and incubated overnight at 37°C. A 81% transmittance cell suspension in Biolog solution IF-0a was then prepared by re-suspending the bacteria grown on BHI agar plates. This suspension was then diluted with Biolog additive solution for the specific plate at manufacturers prescribed ratio. One hundred microliter of the final mixture was then transferred to each of respective PM plate wells. The plates were incubated at 37°C with bacterial active metabolism being read every 15 min for 24 h using Omilog reader (Biolog, Hayward, CA, United States), a system that uses respiration as a universal reporter of active metabolism. Each experiment was performed in duplicate.

### Growth Evaluation

Secondary stationary phase stage BHI cultures prepared as described above from each *L. monocytogenes* strain were diluted in BHI to 10^5^ CFU/ml. To assess growth under NaCl stress, 100 μl volumes of BHI media supplemented with 0 and 16% NaCl were added in triplicate to wells of a 96-well microplate to which 100 μl of the different *L. monocytogenes* strains at 10^5^ CFU/ml had been added. To compare growth in Eagle’s minimum essential medium (MEM), 20 μl of BHI secondary culture from each strain diluted to 10^6^ CFU/ml was added in triplicate to respective wells containing 180 μl of MEM. Cultures were incubated for 24 h at 37°C with shaking in a Synergy HT OD reader (BioTek Instruments, GmbH, Switzerland) and the OD_600_ was measured every 30 min. Maximal growth rates were determined from the OD_600_ growth data using the program DMFit (Baranyi and Roberts, 1994).

### Hemolysis Assays

For hemolysis analysis, sterile filtered 1M dithiothreitol (DTT) (Sigma-Aldrich, Buch, Switzerland) treated supernatants collected from early stationary phase cultures were used. These were collected from cultures grown using overnight cultures to make 1:100 dilutions in pre-heated BHI and incubated at 37°C for 5 h. The cultures were first standardized to the same optical density using BHI and centrifuged to collect the supernatants. To activate the hemolysin, a 1:200 dilution of 1M DTT and each of the supernatant was made and incubated for 1 h at 37°C. One hundred microliter of a 2% washed human red blood cells PBS solution was pipetted to respective 96 well plate wells. Then 100 μl of the DTT activated supernatant was added. The bacterial supernatant and RBC mixture was incubated at 37°C for 40 min to allow hemolysis to occur. After which it was centrifuged for 5 min at 3,100 × *g* and then 100 μl of each lysate was transferred to a new 96 well plate. The degree of hemolysis was assessed by measuring absorbance of the lysate at 420 nm using the Synergy HT OD reader (BioTek Instruments, GmbH, Switzerland). The internal control strains *L. monocytogenes* EGDe and *L. innocua* JF5051 were included in all experiments.

### Cell Invasion Assays

Cell invasion assays were performed in the human enterocyte-like Caco-2 (ATCC^®^ HTB-37^TM^) cell line. Cells were grown to confluence in a 96-well cell culture plate overnight at 37°C, 5% CO_2_ in Eagle’s MEM, (Life Technologies, Switzerland) supplemented with 20% fetal bovine serum. The monolayers were washed with pre-warmed PBS (37°C) and then infected with *L. monocytogenes* strains at a multiplicity of infection (MOI) of 0.01 in MEM. Following 30 min of incubation the medium was removed, then cells were washed with PBS and overlaid with MEM medium containing 0.01 mg/ml gentamicin and incubated for another 60 min at 37°C to kill extracellular bacteria. At the end of the incubation the cells were washed five times with 100 μl warm PBS and then 100 μl of 40 mg/ml saponin was added to lyse the cells. The lysate was serially diluted and cell count was done to determine the number of *L. monocytogenes* recovered in comparison to the number used to infect the Caco-2 cells. Reference strain *L. monocytogenes* EGDe and *L. innocua* JF5051 were included in all experiments as positive and negative controls, respectively.

### Zebra Fish Microinjection Assays

Zebrafish husbandry and assays were performed as previous described (Eshwar et al., 2017) with a few modifications. The *Danio rerio wik* zebrafish line strains were used in this study. All experiments were performed with the approval (no. 216/2012) from the Veterinary Office, Public Health Department, Canton of Zurich (Switzerland). Bacteria for microinjection experiments were harvested from stationary phase BHI bacteria cultures by centrifugation and washing with DPBS, then standardized to the same CFU through plate counts and appropriate dilutions. Two-day post fertilization embryos were injected with approximately 500 CFU in 1–2 nl volume of a bacterial suspension in DPBS into the blood circulation via the caudal vein. The number of CFU injected was determined by direct microinjection of a DPBS droplet on agar plates and confirmed by disintegrating five embryos individually immediately after microinjection and plating the lysates on BHI agar. Post-infection embryos were placed into 24-well plates (one embryo per well) in 1 ml E3 medium per well, incubated at 28°C and observed for signs of disease and survival under a stereomicroscope twice a day. The number of dead larvae was determined visually based on the absence of a heartbeat.

### Genome Analysis

Genomes of *L. monocytogenes* EGDe, LL195, Lm3136, Lm3163, N2306, and N16-0044 are available in GenBank under accession numbers NC003210, HF558398, CP013722, CP013723, CP011004, and CP035187, respectively (Glaser et al., 2001; Weinmaier et al., 2013; Tasara et al., 2015, 2016). Rapid Annotation Subsystem Technology (RAST) and Seed Viewer standard settings^2^ were used for genome annotation and comparisons. Progressive Mauve was used to align the genomes and to derive the coordinates for the positions of the single nucleotide polymorphisms (SNPs), insertions and deletions (InDels) (Darling et al., 2010). Genomes were correlated with PM data using the DuctApe software (Galardini et al., 2014). Only those genes described in the Kyoto Encyclopedia of Genes and Genomes (KEGG) database were considered. Genes found in all strains were described as “core,” and the others as “dispensable”: Dispensable genes were further divided into “accessory,” when a gene is present in at least two strains, and “unique,” when a gene is present in exactly one strain as previously described (Galardini et al., 2014). Genes possibly linked to phenotypic differences were searched and compared between the genomes in CLC genomics Workbench (Qiagen, Prismet, Denmark) and using BLASTn and BLASTp in the National Center for Biotechnology Information (NCBI) platform (blast.ncbi.nlm.nih.gov/Blast.cgi). Relatedness of the strains was assessed by SNP comparisons. SNPs were identified using parsnp within the harvest suite (Treangen et al., 2014) using standard settings and nucleotide fasta files as input. Each strain was used as a reference strain and compared to the other strains. The output files were converted to variant calling files using harvesttools and a SNP matrix was constructed by taking the sum of the variants compared to the reference strain. The SNP matrix was visualized in a heatmap using clustvis (Metsalu and Vilo, 2015). Genome compositions analyses were performed by comparing the protein coding sequences using the script get_homologues (Contreras-Moreira and Vinuesa, 2013). A pangenome was constructed by using get_homologues with the option “-t 0” to obtain all proteins, a cut off of E < 1e-05 for blast searches, and a 75% minimum alignment coverage. Both a cluster of orthologous groups (COG) and an orthologous Markov clustering (OMCL) based pangenome was calculated and only genes presence in both OMCL and COG based pangenome were considered in further pangenome analysis. A presence-absence matrix of the pangenome was produced using the script compare clusters (Contreras-Moreira and Vinuesa, 2013). The pangenome was annotated by selecting the first protein of a cluster and annotating that protein using EggNOG 4.5 (Huerta-Cepas et al., 2016). Virulence genes were identified using get_homologues and the protein database VFDB_SetA_pro from the virulence factor database (Chen et al., 2016) as the reference set. Settings were identical to the settings for the pangenome analyses. The VFDB_SetA_pro contains the experimentally verified virulence factors and was downloaded in November 2018.

### Statistical Analyses

All experiments presented were performed independently at least three times unless stated otherwise. JMP software (Version 12.1.0, SAS Institute Inc., NC, United States) was used for statistical analysis of data. One-way ANOVA with *post hoc* Tukey HSD tests were used to assess statistical significance of differences relative to the reference strains as well as between the strains. *P*-values of <0.05 were considered to be statistically significant. For PM data analysis DuctApe and opm version 1.3.64 software’s were used as previously described (Galardini et al., 2014; Göker et al., 2016). Briefly, for opm based analysis the reference parameter was area under the curve. Whilst for DuctApe the parameter, activity index (AV), was calculated to rank kinetic curves, providing information about the ability to be metabolically active under a specific culture condition. The AV parameter was obtained through k-means clustering on maximum metabolism, area under the curve, average height, lag time, and slope, whilst for opm based analysis k-means clustering was based on area under the curve as previously described (Galardini et al., 2014; Göker et al., 2016). For each compound tested, the final result was expressed as the mean of two replicates. The bacterium was not able to grow under conditions were AV value was equal to zero, whilst it was able to grow under conditions were the AV value was higher than zero.

## Results

### Phenotypic Microarray-Based Comparison of the Studied Strains

**FIGURE 1:**
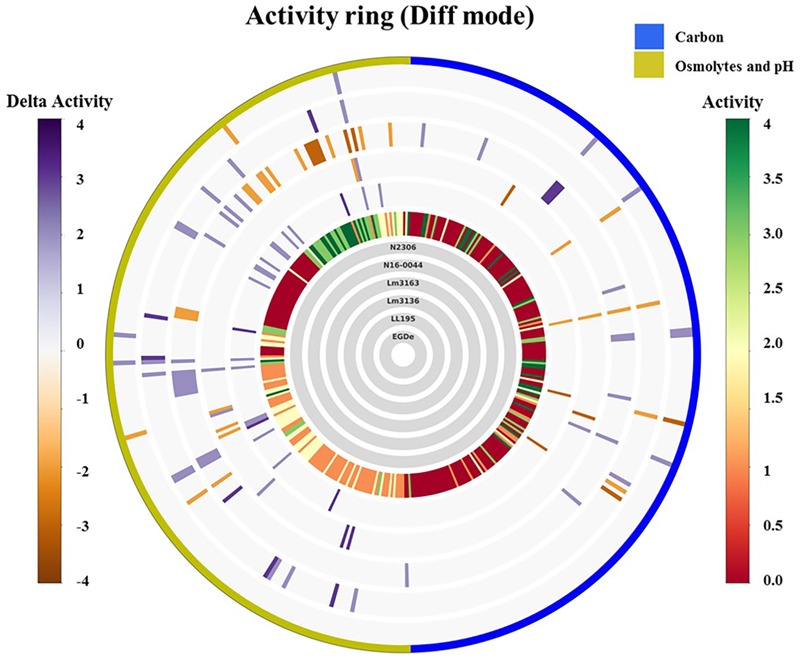
Overall growth/metabolic activity ring based on phenotype microarray comparison of the strains with respect to C-source utilization (PM01 and PM02) and stress (osmolytes and pH; PM09 and PM10) resistance: gray inner circles indicate the strains’ order; external circle indicates the PM categories. The activity index (AV) calculated for each strain and well is reported as color stripes going from red (AV = 0) to green (AV = 4). Delta activity: the difference with the AV value of the reference strain is reported when equal to or higher than 2 AV; gray is no difference; purple indicates a higher activity; orange color indicates a lower activity.

**FIGURE 2:**
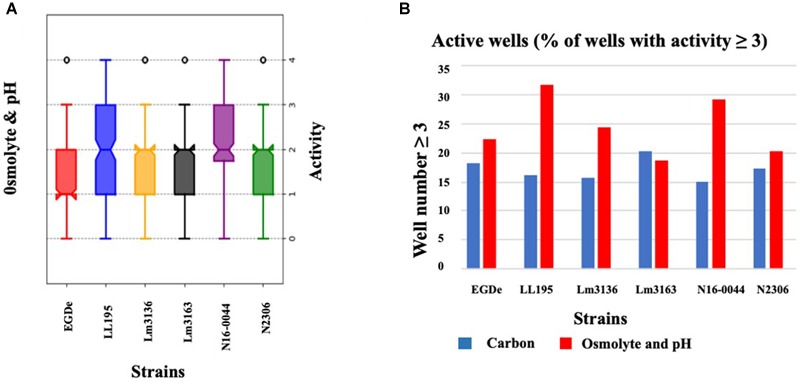
Variation of C-source utilization and stress tolerance profiles among *L. monocytogenes* strains. **(A)** Box-plot showing growth/metabolic activities observed among the study strains under pH and osmotic stress conditions. **(B)** Bar graph displaying the percentage of compounds under which each strain had an activity that equaled or was above 3 AV.

**FIGURE 3:**
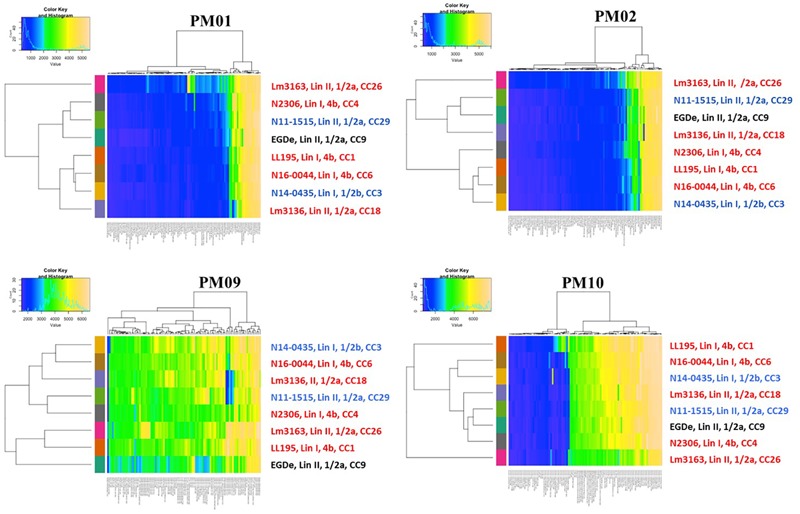
Heatmaps showing clustering of the strains based on PM01, PM02, PM09, and PM10 growth/metabolic activity results. Clinical listeriosis outbreak strains are shown in red, strains isolated from food during routine check are shown in blue and the *L. monocytogenes* EGDe reference strain is shown in black. Each of the colored bars identified on the *x*-axis represents the different PM test conditions applied and for detailed information on the reactions see Supplementary Figures S2A–D.

Using the Biolog phenotypic microarrays, we compared C-source utilization (PM01 and PM02), osmolyte (PM09) and pH (PM10) sensitivity profiles in a set comprising previous listeriosis outbreak and food-associated *L. monocytogenes* strains isolated in Switzerland (Table 1). An overview provided in Figure 1 shows that there were strain-specific differences observed in growth/metabolism activity detected on various C-sources and under different osmolyte and pH stress conditions (Figure 1). The total number of C-sources that were metabolized among the strains ranged from 34 to 51. Interestingly, two strains Lm3136 and Lm3163, that were recovered during the same 2005 Swiss Tomme cheese listeriosis outbreak showed the least and highest numbers of C-sources, respectively, that were metabolized among the tested strains (Table 2). Serotype 4b strains LL195 and N16-0044, that were responsible for the 1983–1987 Vacherin Montd’or cheese and 2016 meat pâté associated listeriosis outbreaks in Switzerland, respectively, were the most stress tolerant with respect to both osmotic and pH stress among the tested strains (Figure 2A). Overall whilst the strain Lm3163 showed highest overall metabolic activity on C-sources, LL195 was the most metabolically active strain under salt and pH stress conditions (Figure 2B). Meanwhile out of the 190 tested C-sources, 31 could be metabolized by all strains whereas another 22 were metabolized by at least one strain (Supplementary Table S1). A comparison of the C-source utilization profiles to those of *L. monocytogenes* EGDe revealed a mixture of metabolic activity capacities among the outbreak strains compared to the reference strain. For example, whilst the EGDe strain did not metabolize D-tagatose both Tomme cheese outbreak associated strains, Lm3136 and Lm3163 could efficiently utilize this C-source (Table 3). Of the 31 C-sources utilized by all strains, 10 C-sources including glycerol, ribose, inosine, maltose, arbutin, and thymidine were metabolized at varying rates by the different strains (Supplementary Figure S1). Meanwhile there were genetic lineage specific trends observed regarding β-D-allose utilization, which was metabolized by all lineage II but not any of the lineage I strains tested (Table 3). Only a single amino acid was utilized as a carbon source by one of the tested strains. Lm3163 was the only strain among the examined strains that utilized L-threonine as a C-source (Table 3 and Supplementary Table S1). An overall attempt to either cluster the strains based on their C-source or the pH and osmotic stress tolerance profiles on the other hand showed that the strains clustered independent of isolation source, serotype and evolutionary genetic lineage (Figure 3 and Supplementary Figures S2A–D).

**Table 2:** Number of C-sources utilized^1^.

Genetic lineage	Lineage I				Lineage II			
Strain ID	LL195	N2306	N16-0044	N14-0435	EGDe	Lm3136	Lm3163	N11-1515
Number of carbon source utilized	35	38	35	35	37	34	51	39

*^*1*^Opm generated table for number of C-sources utilized by each strain.*

**Table 3:** Variation in carbon source utilization among the examined *L. monocytogenes* strains^1^.

PM plate ID	Lineage I				Lineage II			
PM01, PM02	LL195	N2306	N16-0044	N14-0435	EGDe	Lm3136	Lm3163	N11-1515
L-Arabinose	-	-	-	-	-	-	+	-
D-Mannitol	-	-	-	-	-	-	+	-
Lactulose	-	-	-	-	-	-	+	-
Sucrose	-	-	-	-	-	-	+	-
L-Threonine	-	-	-	-	-	-	+	-
Ala-Gly	-	-	-	-	-	-	+	-
Pyruvic acid	-	-	-	-	-	-	+	-
2′-Deoxy-adenosine	-	-	-	-	-	-	+	-
Turanose	-	-	-	-	-	-	+	-
2,3-Butanedione	-	-	-	-	-	-	+	-
Adenosine	-	+	-	-	-	-	-	-
α-D-Lactose	-	+	-	-	-	-	+	-
Pectin	-	-	-	-	-	-	+	+
D-Tagatose	-	-	-	-	-	+	+	-
D-Xylose	+	-	-	-	-	-	+	-
Palatinose	-	+	-	-	+	-	-	+
D-Arabinose	-	-	-	-	-	+	+	+
β-D-Allose	-	-	-	-	+	+	+	+
D-Maltose	-	+	+	+	+	-	+	+
Arbutin	+	+	+	+	+	-	+	+
Inosine	+	+	+	+	+	-	+	+
Thymidine	+	+	+	+	+	-	+	+

*^*1*^C-sources that gave a positive reaction in at least one strain but not all the strains are shown. Symbols: -, negative reaction; +, positive reaction. Opm generated results showing phenotypic variability on different C-sources among the isolates: **(i)** Lm3163 utilizes the most C-sources. **(ii)** Strains demonstrate lineage associated ability to utilize β-*D*-allose.*

#### Outbreak Strains Vary in Metabolism of Host and Food Associated C-Sources

Strain-specific differences in the capacity to utilize some host and food relevant C-sources were also observed among the tested strains (Table 3). Intracellular C-sources nucleosides inosine and thymidine, as well as pyruvic acid and maltose were differentially metabolized by the strains. While Lm3163 was able to utilize all these C-sources, strain Lm3136 isolated from the same listeriosis outbreak was incapable of utilizing most such C-sources (Table 3). Furthermore, differences in metabolic activity on food relevant C-sources such as sucrose, lactose, pectin and D-tagatose were observed, with Lm3163 utilizing all such C-sources whereas most of the tested strains were unable to utilize them (Table 3). Strains Lm3163 and Lm3136 that were isolated from the 2005 Tomme cheese associated listeriosis outbreak were the only strains able to utilize D-tagatose. An ability of these two strains to metabolize a C-source that is often found in dairy products might be suggestive of their adaptation to dairy associated niches linked to milk and cheese.

#### Variation in Osmotic and pH Stress Sensitivity Is Observed Among Outbreak Strains

**FIGURE 4:**
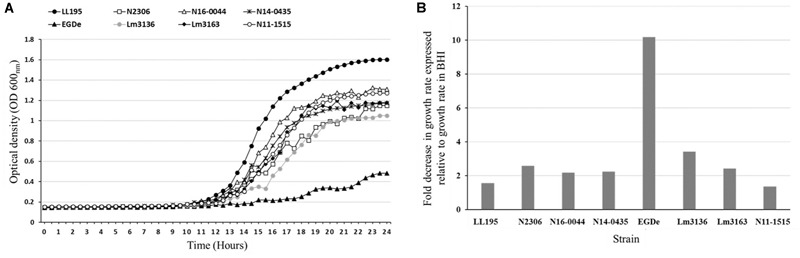
NaCl stress tolerance variation among the *L. monocytogenes* strains. Growth curves **(A)** and bar charts **(B)** showing growth kinetics and fold decrease in growth rate of the *L. monocytogenes* strains during growth in BHI under NaCl salt (8%) stress. LL195 and EGDe strains showed the highest and lowest tolerance to NaCl salt stress, respectively. The data presented represents three independent biological repeats.

All the tested strains gave positive reactions on 92 of the 96 osmotic stress conditions tested on PM09 although they showed variable metabolic rates when exposed to higher concentrations of common food preservatives including NaCl and sodium lactate. At higher NaCl (8–10%) concentrations, the reference strain *L. monocytogenes* EGDe showed the lowest growth/metabolic activity compared to all the other strains (Figure 1, 2). In a validation of these PM observations a growth curve-based comparison showed that while the Swiss Vacherin Montd’or cheese listeriosis outbreak strain LL195 exhibits superior growth, *L. monocytogenes* EGDe was the most impaired strain during growth under salt stress (8% NaCl) in BHI (Figure 4). All tested strains were able to tolerate high concentrations of other common food preservative compounds such as sodium lactate and sodium benzoate (Table 4, Supplementary Table S2, and Supplementary Figure S3). Six strains showed growth/metabolic activity at the highest levels of sodium lactate (12%) and sodium nitrate (100 mM) tested. Strain Lm3136 involved in the Swiss Tomme cheese outbreak as well as N11-1515 isolated from milk in 2014, were the only strains inhibited by 11 and 12% sodium lactate, whereas other strains were not. At 80 mM sodium nitrate, the EGDe reference strain and the other Swiss Tomme cheese associated outbreak strain Lm3136 were inhibited, whilst all the other strains were metabolically active.

**Table 4:** Osmotic and pH stress sensitivity profiles^1^.

PM plate ID	Lineage I				Lineage II			
PM09	LL195	N2306	N16-0044	N14-04354	EGDe	Lm3136	Lm3163	N11-1515
10% NaCl	+	+	+	+	+	+	+	+
11% Sodium lactate	+	+	+	+	+	-	+	-
200 mM sodium benzoate + pH 5.2	+	+	+	+	+	+	+	+
80 mM sodium nitrite	+	+	+	+	-	-	+	+
**PM10**								
pH 4.5	-	-	-	-	-	-	-	-
L-Asparagine + pH 4.5	+	-	-	-	-	-	-	-
L-Serine + pH 4.5	+	-	-	-	-	-	-	-
L-Citrulline + pH 4.5	-	-	+	-	-	-	-	-
L-Valine + pH 4.5	+	-	+	-	-	-	-	-
L-Hydroxyproline + pH 4.5	+	-	+	-	-	-	+	-
L-Ornithine + pH 4.5	-	-	-	+	-	-	-	-
L-Homoserine + pH 4.5	+	-	-	-	-	-	-	-
L-Norvaline + pH 4.5	+	+	+	+	+	+	+	+
D,L-α-Amino-butyric acid + pH 4.5	+	-	-	-	-	-	+	-
L-Cysteic acid + pH 4.5	+	-	+	-	-	-	+	-
5-Hydroxy-L-lysine + pH 4.5	+	-	+	-	-	-	-	-
D,L-Diamino-pimelic acid + pH 4.5	-	-	-	-	-	-	+	-
Urea + pH 4.5	+	-	-	-	-	-	-	-
pH 9.5	+	+	+	+	+	+	+	+
β-Phenylethylamine + pH 9.5	+	+	+	+	+	-	-	+

*^*1*^Opm generated results showing phenotypic variability on different osmotic and pH stress conditions among the isolates: Substances such as *L*-norvaline enhanced *L. monocytogenes* resistance to acidic stress whereas β-phenylethylamine inhibited alkaline stress resistance in a strain dependent manner. Symbols: -, negative reaction; +, positive reaction.*

Out of the 96 pH conditions tested on PM10 a positive growth/metabolic activity reaction for at least one strain was detected in 71 assay conditions. The Vacherin Montd’or cheese associated outbreak strain LL195 generated the highest number of positive reactions under the tested range of pH stress conditions (Figure 1, 2, Table 4, and Supplementary Table S3). While all the tested strains gave negative results for active growth/metabolic activity at pH 4.5 and below, the inclusion of the compound L-norvaline enhanced their acidic pH tolerance allowing all the tested strains to grow or become metabolically active at pH 4.5 (Table 4 and Supplementary Table S3). Supplementation with some amino acids gave positive growth/metabolic activity reactions under acid stress at pH 4.5 with some but not all the tested strains indicating the existence of strain-specific differences in the capacity to use some amino acids for acid stress tolerance. LL195, N16-0044, and Lm3163 were for example able to grow at pH 4.5 when supplemented with L-hydroxyproline while the rest of the strains could not. LL195 was the only strain showing active growth/metabolism at pH 4.5 in presence of urea indicating urease dependent acid stress tolerance activity in this strain. Meanwhile all strains gave positive growth/metabolism reactions at pH 5–10 but there were strain associated differences observed in metabolic rates under such conditions (Supplementary Table S3). At pH 9.5 there was an inhibition of Lm3136 and Lm3163 strains detected in presence of β-phenylethylamine while the rest of the tested strains were not affected. None of the amino acids altering *L. monocytogenes* pH sensitivity were directly utilized as C-sources on PM01 and PM02 in the examined strains suggesting that the protective effects of these amino acids might not involve their metabolism as C-sources. Overall, the two lineage I and serotype 4b outbreak strains LL195 (1983–1987 Vacherin Montd’or cheese outbreak) and N16-0044 (Meat pâté outbreak) showed the greatest tolerance to both pH and osmotic stress in comparison to all the other tested strains (Figure 1, 2). Furthermore, the studied strains were able to metabolize all the chromogenic compounds tested on PM10, suggesting that these substances could be used in chromogenic *L. monocytogenes* detection or isolation media.

### Outbreak Associated Strains Vary in Cell Invasion and Zebrafish Virulence

**FIGURE 5:**
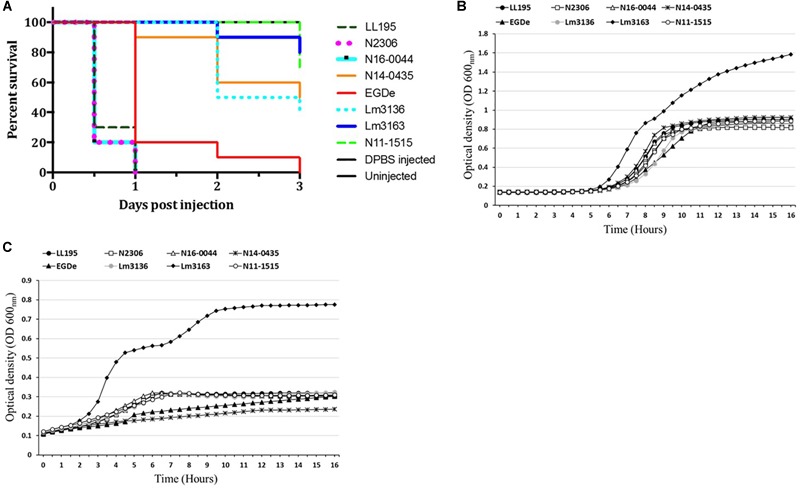
Strains vary in virulence and growth in BHI and MEM media. **(A)** Virulence was assessed using a zebrafish infection model. Zebrafish embryos (*n* = 10 per strain) infected (500 CFU) with different *Listeria monocytogenes* strains were monitored over 3 days. Zebra fish embryos more rapidly succumbed to serotype 4b compared to serotype 1/2a strains. Kinetic growth assays in **(B)** BHI and **(C)** MEM based on OD_600_ measurement showing varied growth of the examined *L. monocytogenes* strains with Lm3163 displaying the greatest growth capacity and biphasic growth in both media compared to the other strains.

In order to examine if C-source metabolism and stress resistance variations could be related to pathogenicity we compared virulence between the Swiss listeriosis outbreak strains and the reference strain *L. monocytogenes* EGDe. Listeriosis outbreak strains showed significantly higher (*P* < 0.05) cell invasion (29- to 504-fold) capacities compared to *L. monocytogenes* EGDe when examined using the Caco-2 cell-based infection model (Table 5). Notably Lm3163 displaying highest levels of metabolic activity and C-source substrate diversity also showed highest cellular invasion levels compared to all other tested strains (Table 5). Comparison of strain virulence *in vivo* in a zebrafish embryo-based infection model further revealed strain specific variation in virulence based on the rate and mortality levels achieved. Lineage I, serotype 4b outbreak strains (LL195, N16-0044, N2306) caused significantly higher mortality compared to the lineage II, serotype 1/2a outbreak strains (Lm3136 and Lm3163) and *L. monocytogenes* EGDe (Figure 5A). In contrast, strain Lm3163 despite exhibiting a more diverse C-source metabolic capacity and high cell invasion capacity caused the lowest mortality than all other tested strains in this infection model (Figure 5A). *L. monocytogenes* EGDe despite exhibiting the lowest cell invasion levels in Caco-2 cells induced the highest levels of zebrafish embryo mortality compared to the lineage II outbreak strains Lm3136 and Lm3163. A comparison of hemolysis among the strains, however, did not show significant differences in hemolytic activity among the examined strains (data not shown). This observation rules out that differences in expression of this virulence function as a reason for the strain-specific differences observed in both cell invasion and zebrafish virulence. A possible explanation for the high invasiveness observed for Lm3163 could in part be due to the fact that this strain due to its high and diverse C-source metabolic capacity might outgrow other strains during incubation in MEM prior to invasion and inside cells post invasion. A notion supported by the observation that this strain grows better than all the other tested strains in BHI and MEM (Figure 5B,C). Another interesting observation is that in both media this strain exhibits a biphasic growth profile typical of changes in utilization efficiency of available nutrients such as C-sources as growth incubation time progresses.

**Table 5:** Comparison of Caco-2 cell invasion capacities.

Strain ID	Percentage Caco-2 cell invasion	Fold invasion expressed relative to *L. monocytogenes* EGDe
**Lineage I**		
LL195	0.0365 ± 0.0080	29.91
N2306	0.01475 ± 0.0065	12.07
N16-0044	0.0211 ± 0.0158	17.27
**Lineage II**		
EGDe	0.0012 ± 0.0004	1
Lm3136	0.0055 ± 0.0032	4.53
Lm3163	0.6161 ± 0.3133	504.30

### Linking Phenotypic Variation to Genomic Differences Amongst the Listeriosis Outbreak Strains

**FIGURE 6:**
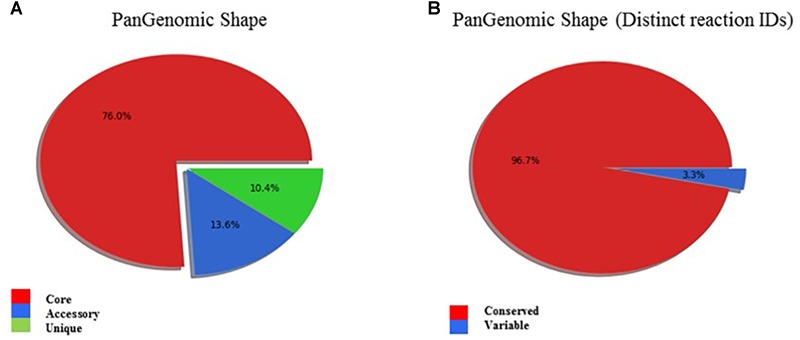
**(A)** PanGenomic shape showing high genomic conservation amongst outbreak associated *L. monocytogenes* strains. Genes found in all strains are labeled as “**core**,” and the others as “**dispensable**”: Dispensable genome is divided into “**accessory**,” when a gene is present in at least two strains, and “**unique**,” when a gene is present in exactly one strain. **(B)** PanGenomic Shape based on distinct reaction IDs demonstrates high metabolic pathway conservation amongst the outbreak associated *L. monocytogenes* strains.

A genome and phenome-based analysis linking variation in C-source metabolism phenotypes to the genomic information of the listeriosis outbreak strains was conducted using the program DuctApe. A comparison of the pangenome and metabolic pathway constructions revealed high conservation between genomes (76% conservation) and metabolic pathways in the examined outbreak strains and the *L. monocytogenes* EGDe reference strain (Figure 6 and Table 6). An observation suggesting that phenotypic variations observed between the outbreak strains in C-source metabolism phenotypes could be a function of minor differences affecting genes and metabolic pathways. A gene content comparison identified 81 lineage specific gene content differences that includes genes associated with transport, metabolism, transcription regulation, virulence, and cell envelope modification (Supplementary Table S4). One such gene content variation could be linked to D-allose metabolism differences detected between the genetic lineages. The genes of the *lmo0734*-*0739* cassette encoding proteins involved in D-allose metabolism are found in genetic lineage II but not genetic lineage I listeriosis outbreak strains of our study (Supplementary Table S4). Consequently, all lineage I outbreak strains examined were unable to use D-allose as a C-source. Presence of numerous genes involved in the transport and metabolism of simple sugars, complex carbohydrates, amino acids and peptides was confirmed in the genomes of the examined strains (data not shown). In some cases, however, the number and composition of these nutrient transporters vary in a lineage and strain specific manner. A lineage specific trend in the distribution of several ATP-binding cassette (ABC transporters) and PTS transporter systems was detected (Supplementary Table S4). Notably one of such differences is associated with the lineage II specific D-allose metabolism as mentioned above. Overall a one-to-one assignment of nutrient and corresponding gene association in most cases was not always possible since the majority of C-sources tested have more than a single gene associated with their transport or metabolism. In another observation there were some C-sources that were metabolized but a complete pathway for their metabolism was not detected in the genome of the examined strains. The strain Lm3163 for example metabolized sucrose but no complete pathway for sucrose metabolism was detected in this strain based on the current genome annotations of *L. monocytogenes* EGDe applied as reference.

**Table 6:** Genome analysis^1^.

		Mapped to	KEGG			Unique	Exclusive
Kind	Size	KEGG	orthology IDs	Pathways	Reactions	reactions	reaction IDs
Core	2503	1576	1260	108	1381	891	786
Dispensable	1032	191	159	58	153	135	30
Accessory	651	126	107	42	106	97	27
Unique	381	65	61	37	47	42	1

*^*1*^DuctApe derived genome analysis statistics, the exclusive reaction indicated is reaction 3.2.1.122 only detected in strain N2306. Only half of the genes of each genome were mapped to KEGG. Genes found in all strains are described as “core,” the other “dispensable”: Dispensable genome is further divided into “accessory,” when a gene is present in at least two strains, and “unique,” when a gene is present in exactly one strain.*

**FIGURE 7:**
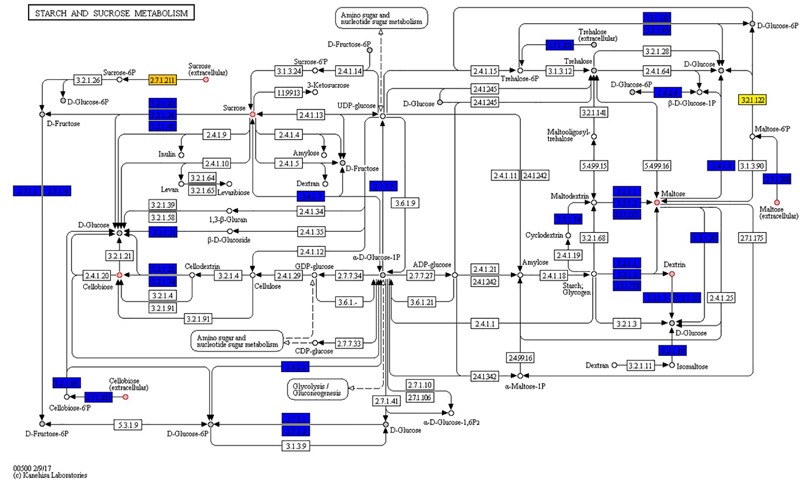
Strains show high phenotypic variability despite high genomic and metabolic pathway conservations. Starch and sucrose metabolism map: boxes represent reactions while circles represent compounds. Core reactions are colored blue, variable reactions are colored orange or yellow; compounds present in PM plates are filled with gray. Red circles around compounds highlight those compounds for which at least one strain has an AV difference with another strain equal or higher than 2 AV.

Genome and phenome analysis also revealed various metabolic pathways where there was genetic and phenotypic variability among the strains leading in some cases to the identification of single reactions that were responsible for the observed metabolic variability. Examples include the starch and sucrose metabolism pathway shown in Figure 7. As indicated by the boxes highlighted in orange and yellow in Figure 7 there were variations associated with the presence of sucrose PTS permease (reaction 2.7.1.211) and maltose-6′-phosphate 6-phosphoglucohydrolase (reaction 3.2.1.122) proteins that are encoded by genes constituting the dispensable genome among strains. Genes encoding for the 2.7.1.211 protein were only detected in LL195, N16-0044, and N2306 strains whereas the 3.2.1.122 protein encoding gene was only detected in N2306. Notably, the latter also represents the only unique reaction identified by DuctApe amongst the studied strains (Table 6). The other C-sources also found to have a high phenotypic variability in their metabolism were cellobiose, dextrin and maltose (Figure 7). The analysis also indicated that most of the reactions are catalyzed by proteins encoded by genes which were not retrieved from the genomic data (Figure 6 and Table 6). The largest source of variation in genome content between the examined strains was related to the number and composition of the prophage elements (Supplementary Table S4). Although there are some strain specific differences, outbreak strains of lineage II contained more phage encoded elements than lineage I strains. The possible influence of such variations in these phage elements on C-source metabolism as well as stress response and virulence phenotypes observed here presently remains unknown.

**FIGURE 8:**
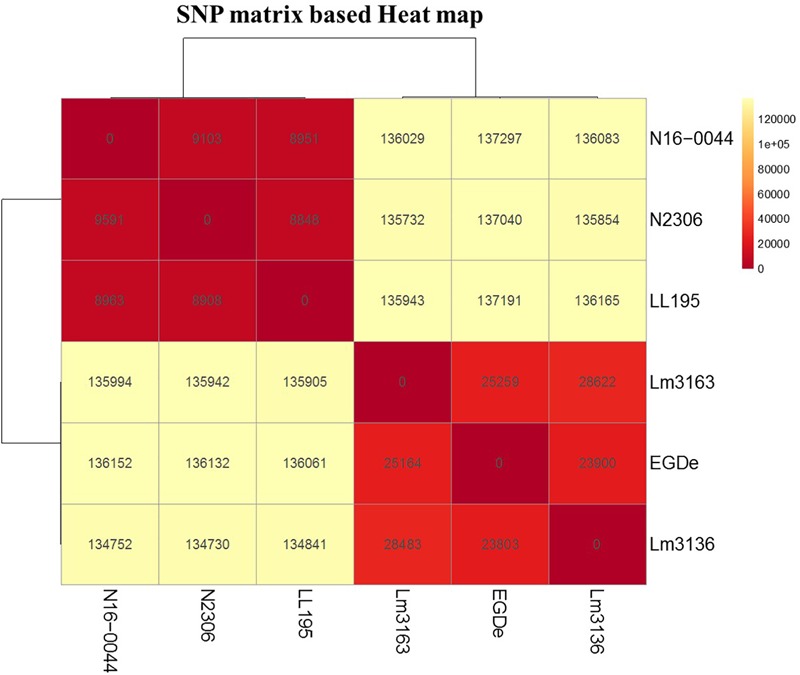
SNP matrix-based heatmap showing the variation in core genome SNPs between strains examined in this study.

Meanwhile there were also several thousands (8,908–137,297 SNPs) of SNP differences detected between the studied outbreak strains (Figure 8). It is thus possible that some of these SNP differences are functionally relevant and contribute to differences observed not only in C-source utilization phenotypes but also to variations in stress resistance and virulence phenotypes that were detected between this set of listeriosis outbreak strains. A comparison with respect to genome virulence factor composition and sequence among the strains also revealed that despite high conservation in most virulence factors there were various minor lineage and strain specific genetic differences detected in some of the key virulence factors including some amino acid changing SNPs in internalin A (Supplementary Tables S5, S6). Therefore, our observation in this regard indicates that the differences observed in host cell invasion and virulence capacity amongst the examined outbreak strains might involve variation in a few genes and/or differences in overall virulence gene regulation and expression between strains. Uncovering such differences, however, will require further investigation in these strains in the future.

## Discussion

In this study, we describe the comparative analysis in a set of clinical and food related *L. monocytogenes* strains including those responsible for previous Swiss listeriosis outbreaks. Despite high levels of synteny in gene content and conservation of metabolic pathways our analysis uncovered significant phenotypic differences among strains with respect to metabolism of host cell and food relevant C-sources as well as resistance of some common food preservatives. Strain Lm3163 that caused the 2005 Tomme cheese Swiss listeriosis outbreak exhibited highest C-source metabolic activity and versatility among the tested strains. It efficiently utilized several food and host relevant C-sources that other tested strains could not metabolize including lactose, sucrose, pyruvic acid and nucleosides. All in all, the examined strain set varied with respect to C-source utilization, osmotic and pH stress tolerance, virulence capacity, and growth in nutrient rich bacterial and cell culture media. The various phenotypic diversities uncovered could provide selective advantages to some of the strains in natural and food-associated environments leading to increased survival and expanded niche range for such strains in the environment as well as infected hosts. Genome sequence comparison in outbreak associated strains revealed minor gene content differences between the strains including lineage specific distribution of some genes. One notable such genetic variation with an impact on C-source utilization involves the D-allose metabolism associated *lmo0734* to *lmo0739* gene cassette found in genetic lineage II but not in lineage I strains. Similar observations were reported from the examination of an even larger *L. monocytogenes* strain collection from China (Liu et al., 2017). Overall our observations indicate that minor genetic changes possibly induced through SNPs as well as variations in gene expression regulation amongst strains are probably behind the bulk of the phenotypic differences detected in our study.

*Listeria monocytogenes* has evolved to survive and replicate in the cytosol of eukaryotic cells including specific metabolic adaptations to optimize survival of host-derived stresses while promoting nutrient acquisition and proliferation within host cells (Grubmüller et al., 2014; Chen et al., 2017). Glycerol and glucose-6-phosphate play an important role as major intracellular carbon substrates for energy generation and anabolism respectively for this bacterium (Grubmüller et al., 2014). In agreement with this all analyzed strains used glycerol but not glucose-6P as a C-source. Despite presence of complete glycolytic and pentose-phosphate pathways based on genome analysis these strains were unable to utilize glucose-6P. This is consistent with repressed uptake of this C-source under extracellular assay conditions as applied in our study (Kreft and Vazquez-Boland, 2001). This repression arises from extracellular suppression of *prfA* expression, which positively regulates the expression of the *hpt* gene encoding the Hpt transporter required for the uptake of glucose-6P (Chico-Calero et al., 2002). Most of the amino acids imported by *L. monocytogenes* inside host cells are directly used for protein biosynthesis and are hardly catabolized (Grubmüller et al., 2014; Chen et al., 2017). Consistent with such observations the examined strains only used a single amino acid Threonine as a carbon source, albeit at a slow rate. Previous work on the EGDe strain suggested that *L. monocytogenes* is not able to utilize pyruvic acid (Grubmüller et al., 2014). While most examined strains could not utilize pyruvic acid as a C-source, Lm3163 was an exception it was able to use it as a C-source.

Survival and adaptation against acidic stress is crucial for host pathogenicity since *L. monocytogenes* encounters acidic challenge in the gastrointestinal tract and phagocytic vacuole during infection (Sleator and Hill, 2002; Begley and Hill, 2015). Observed differences in pH stress sensitivity amongst strains indicate differences in acid stress response mechanisms or their functional efficiencies in examined strains. Variable acid stress adaptation capacities among the examined strains could also have an impact on host pathogenicity since some acid tolerance systems are also linked to virulence in *L. monocytogenes* (Bowman et al., 2010; Begley and Hill, 2015). Serovars specific differences in distribution of acid stress survival genes such as those of the *gadD1T1* operon among non-serotype four strains have been reported (Cotter and Hill, 2003). In our case, however, serotype 4b outbreak strains LL195 and N16-0044 showed higher acid stress tolerance than others indicating the contribution of other mechanism besides *gadD1T1* operon to observed acid stress tolerance differences. Previous studies by others also noted increased organic acid stress exposure tolerance in some outbreak related serotype 4b isolates but mechanisms underlying such an enhanced acid tolerance among the serotype 4b strains remain unknown (Lianou et al., 2006).

Our findings here also showed that the availability of some amino acids enhances *L. monocytogenes* resistance to acidic pH (Table 4). Growth at pH 4.5 was restored in all tested strains in presence of L-norvaline suggesting that this amino acid enhances acidic pH stress resistance in *L. monocytogenes*. Protective mechanisms underlying such a phenomenon are not yet clear, but it appears to be not connected with an ability to metabolize this amino acid. Inclusion of β-phenylethylamine on the other hand inhibited growth/metabolism in strains Lm3136 and Lm3163 at pH 9.5, indicating a strain specific ability of this amino acid to inhibit *L. monocytogenes* growth under alkaline conditions. Interestingly, similar observations were reported in *E. coli* where β-phenylethylamine was used to reduce its growth on meat and biofilm production (Lynnes et al., 2014). Our observations suggest that β-phenylethylamine can also potentially be exploited in developing novel ways to control *L. monocytogenes* in foods.

Osmolytes including NaCl are widely used as food preservatives against bacteria and *L. monocytogenes* encounters osmotic stress within human gastrointestinal tracts (Bergholz et al., 2010; Schuppler and Loessner, 2010; Begley and Hill, 2015). Increased osmotic stress tolerance can thus improve survival of this pathogen in environmental niches associated with foods and human gastrointestinal tracts. As most of our study strains were associated with foods where elevated salt concentrations are encountered, their enhanced osmo-tolerance properties could have been an important factor in enabling survival and growth at elevated salt concentrations in such foods. An association between salt stress adaptation and virulence in *L. monocytogenes* was previously highlighted and an osmotic stress sensitive mutant deleted in the osmotic stress protection gene *opuC* showed poor colonization of the upper small intestine and a reduced ability to cause systemic infection in mice (Sleator et al., 2001; Begley and Hill, 2015). We presume that some outbreak strains in our study showing high tolerance to elevated NaCl salt levels could be better equipped for survival and growth in food as well as in subsequent human gastrointestinal colonization and infection. Strain LL195 isolated during the 1983–1987 Vacherin Montd’or cheese associated listeriosis outbreak showed enhanced salt and acid stress tolerance. Such features could have aided in long-term survival and proliferation of this strain in cheese and associated processing environment where both salt and acid stress conditions are encountered. The strains LL195 and N16-0044 that showed the highest acid and osmotic stress tolerance were also the most pathogenic strains as evaluated in the zebrafish infection model.

Our study also uncovered variable growth among some of the examined strains even in nutrient rich media such as BHI and MEM. The Tomme cheese outbreak-associated strain Lm3163 grew more rapidly than all the other strains and showed biphasic growth curves in both media (Figure 5). One possible explanation for the biphasic growth curves could therefore be the strain switching carbon source utilization during growth when one of the more highly metabolizable source gets exhausted. This effect might be explained through the carbon catabolite repression (Kreft and Vazquez-Boland, 2001). As earlier indicated, this strain has an expanded carbon source metabolic profile. When a strain achieves such increased capacity to utilize a wide spectrum of nutrient sources, it is more likely to grow rapidly on contaminated food. This might also explain why this strain was isolated during an outbreak where CFU counts as high as 3.2 × 10^4^ CFU/g were observed on the contaminated cheese (Bille et al., 2006). Although it might appear trivial, the ability of this strains to use some nutrients such as xylose and pectin could also have had a profound effect on survival and dissemination in the environment.

During infection, *L. monocytogenes* re-encounters many of the same stresses it experiences in food matrices meaning mechanisms essential for survival in foods can also influence host virulence potential. Certain environmental conditions such as exposure to mild acid stress might adapt foodborne pathogens to life within the host (Conte et al., 2000; Begley and Hill, 2015). In a bid to assess for possible relationship between the phenotypic diversity uncovered in carbon source metabolism and stress resistance to host pathogenicity we also evaluated our strains with respect to their virulence phenotypes. *L. monocytogenes* virulence and survival within host cells is dependent on a variety of virulence proteins including the hemolysin listeriolysin O (LLO) that are regulated through PrfA (Chatterjee et al., 2006; Desvaux and Hebraud, 2006; Cossart and Lebreton, 2014; Radoshevich and Cossart, 2018). Despite LLO production as judged by hemolysis levels being similar among strains, listeriosis outbreak strains showed superior epithelial cell invasion ability than the reference strain *L. monocytogenes* EGDe. The cell invasion capacity of Lm3163 was significantly higher compared to all the other tested strains. We hypothesize that the high invasiveness of this strain might be due to its ability to multiply more rapidly than other strains within the cell culture media and infected cells. Lm3163 has an expanded and more flexible metabolic profile that includes metabolism of intracellular located substrates such as pyruvic acid suggesting that it might have multiplied faster during the invasion assay in comparison to the other strains in the media and inside the cell after invasion. In addition, there were amino acid changing SNPs detected in some virulence factors including Internalin A, which could also have contributed to host cell invasion differences observed between strains.

*Listeria monocytogenes* strains vary in virulence capacity as indicated by the variable distribution of genetic and serological subtypes in food associated environments compared to human and animal clinical listeriosis cases (Liu, 2008; Orsi et al., 2011; Maury et al., 2016; Moura et al., 2016). Virulence analysis using zebrafish embryos indicated genetic lineage and serotype related trends with lineage I and serotype 4b strains being more virulent than lineage II and serotype 1/2a strains. Interestingly Lm3163 the strain that utilized most of the C-sources while highly invasive in Caco-2 cells was the least virulent in this model. Reasons for this remain unknown although it might be tempting to speculate that its ability to metabolize some C-sources found in the zebrafish host environment could also have a repressive effect on virulence gene expression. It is known that metabolism of some carbohydrates taken up via the PTS strongly repress the expression of virulence genes (Deutscher et al., 2014). PrfA, the main regulator of virulence genes is repressed by some PTS substrates although mechanisms of such repressions are not yet fully understood (de las Heras et al., 2011; Cossart and Lebreton, 2014; Deutscher et al., 2014; Radoshevich and Cossart, 2018). Notably Lm3163 was the only strain that could metabolize PTS sugars such as mannitol, lactose, and sucrose, which could in part have contributed to virulence repression in this strain (Kreft and Vazquez-Boland, 2001). Although genome virulence factor composition analysis showed a high conservation of most virulence factors there were also some lineage specific virulence genes detected. Such lineage specific virulence gene distribution coupled with some amino acid changing SNPs in conserved virulence genes and differences gene expression regulation could all contribute to observed lineage associated strain variability in pathogenicity.

Genome comparisons between the strains showed high genome conservation consistent with previous observations among *L. monocytogenes* strains (Orsi et al., 2011). Despite this some minor differences that could contribute to phenotypic differences amongst strains were observed including the lineage specific distribution of the *lmo0734* to *lmo0739* gene cassette in lineage II but not lineage I strains, which explains lineage specific difference in allose metabolism as described recently (Zhang et al., 2018). This could in part explain the predominance of lineage II amongst food isolates when isolation media that employ D-allose supplemented enrichment broths are used to improve *L. monocytogenes* isolation and reduce the growth of non-target organisms (Liu et al., 2017). The *araBAD* operon that directs arabinose catabolism in *E. coli* is dynamically activated in the presence of arabinose and the absence of glucose (Desai and Rao, 2010). Lm3163 utilized both D- and L-arabinose whilst the Lm3136 and N11-1515 strains utilized the former suggesting that they might possess a similar catabolic mechanism. Isolation and identification protocols often describe *L. monocytogenes* as incapable of utilizing L-arabinose and D-mannitol (Gawade et al., 2011; Orsi and Wiedmann, 2016), the demonstration that Lm3163 can metabolize both sugars bring into question whether some isolates could be miss classified as non-*L. monocytogenes*. Genomes of the study strains also contain varying numbers and composition of phage-related elements with lineage II containing more elements than lineage I strains. Prophages have been demonstrated to both detract from and add to an organism’s fitness, by disrupting the function of the gene into which they have incorporated or, by encoding genes aiding faster cell growth (Milillo et al., 2012). It is thus plausible that some of these elements might be contributing to phenotypic variability observed amongst strains with respect to growth capacity and virulence phenotypes.

Phenome genome link analysis with DuctApe revealed some metabolic pathways showing genetic and phenotypic variability among the analyzed strains. It revealed that some alternate pathways for certain carbon source metabolism might exist and are yet to be discovered. This is exemplified by the starch and sucrose metabolism pathway, which highlighted that Lm3163 lacks a transporter (sucrose PTS permease) required for sucrose uptake but still metabolizes sucrose. Whereas LL195, N16-0044, and N2306 strains carrying genes encoding the sucrose transporter could not utilize sucrose. It also revealed that strains with similar cellobiose metabolism gene content patterns had different cellobiose metabolism capacities a phenomenon that might be a result of epigenetics. It also highlighted the presence of genes encoding maltose-6′-phosphate 6-phosphoglucohydrolase on N2306 genome. However, the importance of this *Listeria* pathogenicity island 4 (LIP4) associated protein regarding maltose metabolism could not be established as this strain had similar maltose metabolic capacity like other strains lacking this enzyme (Maury et al., 2016). Two strains could not utilize maltose despite having complete pathways for its metabolism, which might also be attributed to differences in the epigenetic regulation of gene expression among strains. Meanwhile the differences in genetic makeup amongst the strains and between the lineages such as the variability in number and composition of transporters and pumps amongst the strains and between lineages could in some cases be associated with lineage and strain specific phenotypic differences such as lineage II specific D-allose metabolism or the ability of Lm3163 to utilize the most diverse set of C-sources.

All bacteria require nutrients and minerals for growth. For saprophytic or pathogenic bacterium, these nutrients can be derived from decaying vegetation, host body fluids, host cells or other resident or dietary microbes (Fischbach and Sonnenburg, 2011). Knowing the full metabolic requirements of a bacterium can lead to a better understanding of the conditions under which it is likely to thrive in food vehicles and infected hosts as well as enable the design of interventions to prevent this. Overall the C-source utilization and stress resistance profiles defined might provide a basis for developing improved media for *L. monocytogenes* isolation and detection. Alternatively, such knowledge can also be exploited to come up with novel protocols for *L. monocytogenes* control in foods, for example, by limiting nutrient availability through the use of natural inhibitors or chemical mimics that block uptake of certain key C-sources. This data forms the basis for further studies of such nature with more strains to understand the extent of these phenotypic differences and possibly apply the knowledge gained to improve food safety.

## Author Contributions

TT designed and supervised the study. FM and AE performed the experiments. UvA assisted with the PM experiments. FM, TT, AE, UvA, and MS analyzed the data and wrote the manuscript.

## Conflict of Interest Statement

The authors declare that the research was conducted in the absence of any commercial or financial relationships that could be construed as a potential conflict of interest.

## Footnotes

1 https://biolog.com

2 http://rast.nmpdr.org
