# Supplementary material for: Variable Carbon Source Utilization, Stress Resistance, and Virulence Profiles Among Listeria monocytogenes Strains Responsible for Listeriosis Outbreaks in Switzerland
Source: Front Microbiol. 2019 May 3;10:957. doi: 10.3389/fmicb.2019.00957 (PMC6510287; doi:10.3389/fmicb.2019.00957)

Figure S1. Respiration rate based kinetic growth assays showing that *Listeria monocytogenes* strains displayed varied metabolic rates on selected carbon sources.

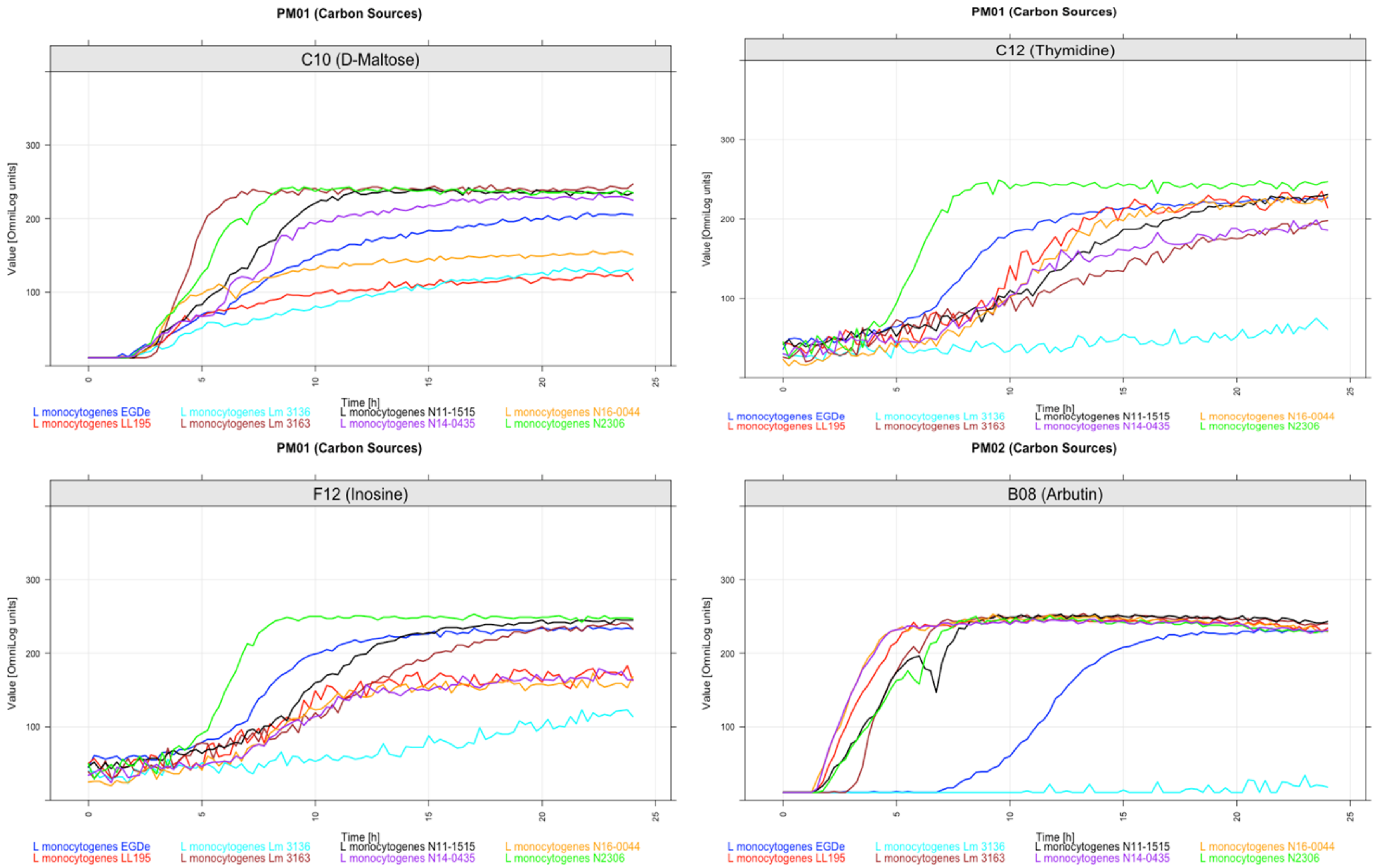

Figure S2A. Heat map showing clustering of the strains based on PM01 growth/metabolic activity results. Clinical listeriosis outbreak strains are shown in red, strains isolated from food during routine check are shown in blue and the *L. monocytogenes* EGDe reference strain is shown in black.

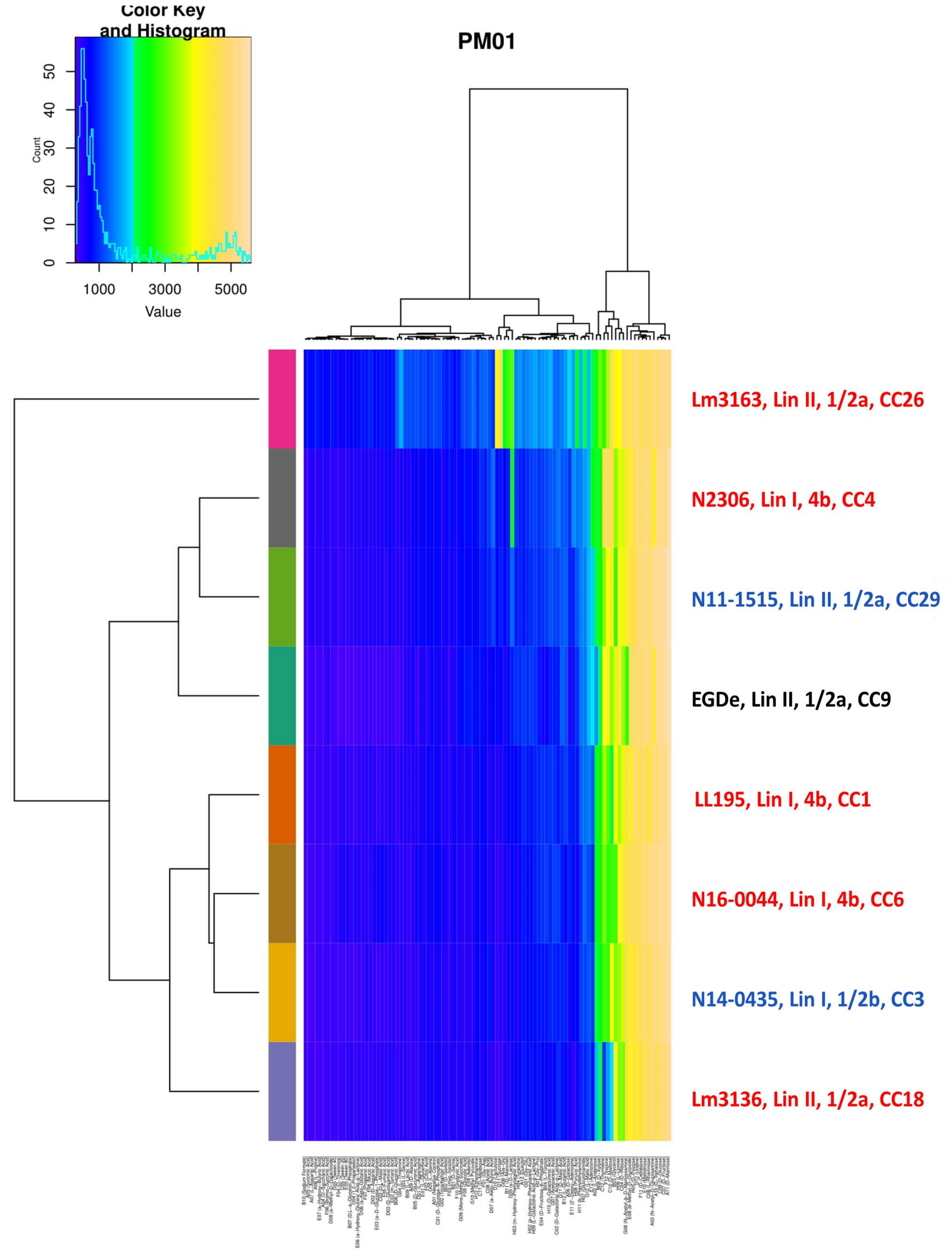

Figure S2B. Heat map showing clustering of the strains based on PM02 growth/metabolic activity results. Clinical listeriosis outbreak strains are shown in red, strains isolated from food during routine check are shown in blue and the *L. monocytogenes* EGDe reference strain is shown in black.

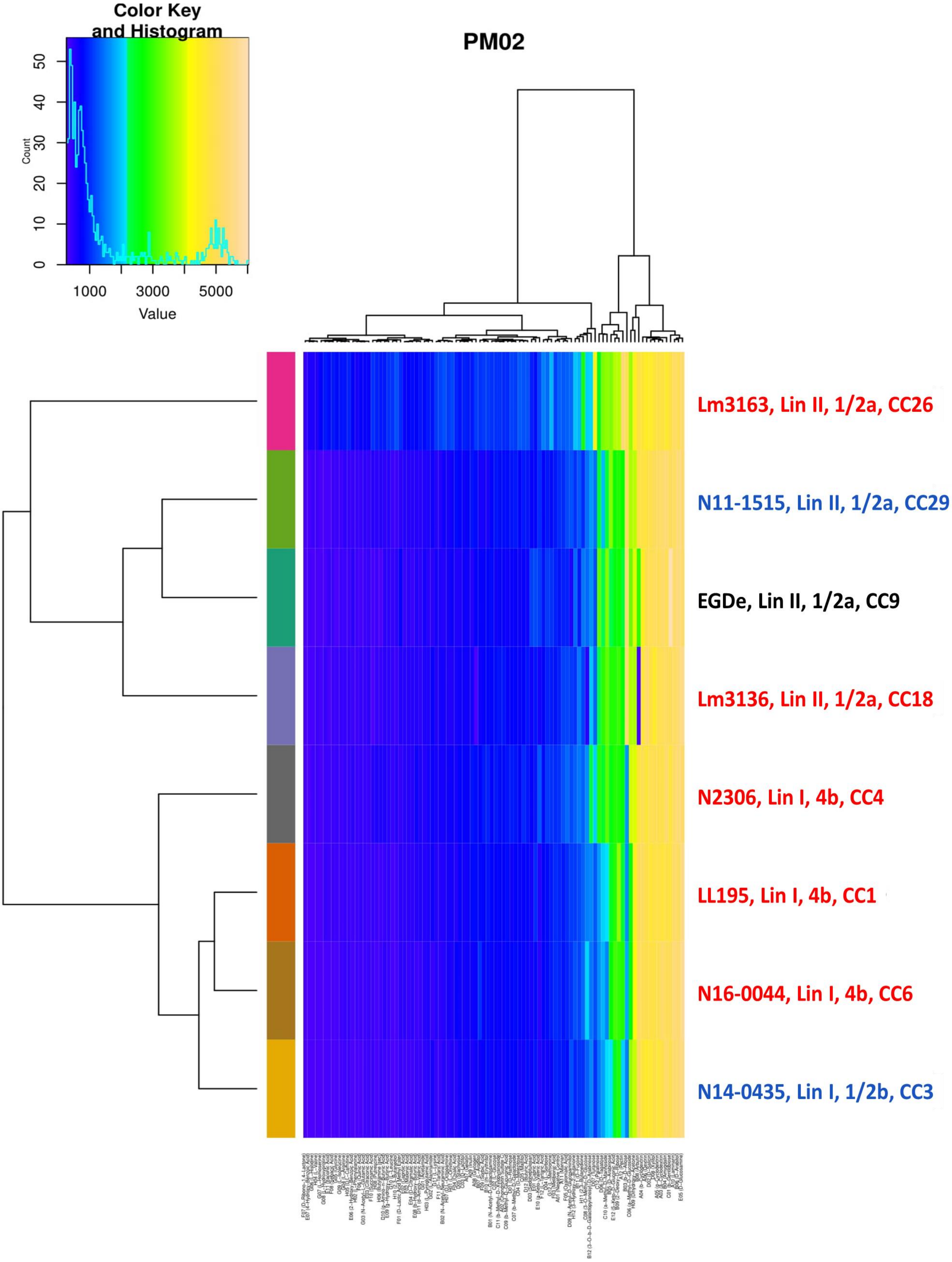

Figure S2C. Heat map showing clustering of the strains based on PM09 growth/metabolic activity results. Clinical listeriosis outbreak strains are shown in red, strains isolated from food during routine check are shown in blue and the *L. monocytogenes* EGDe reference strain is shown in black.

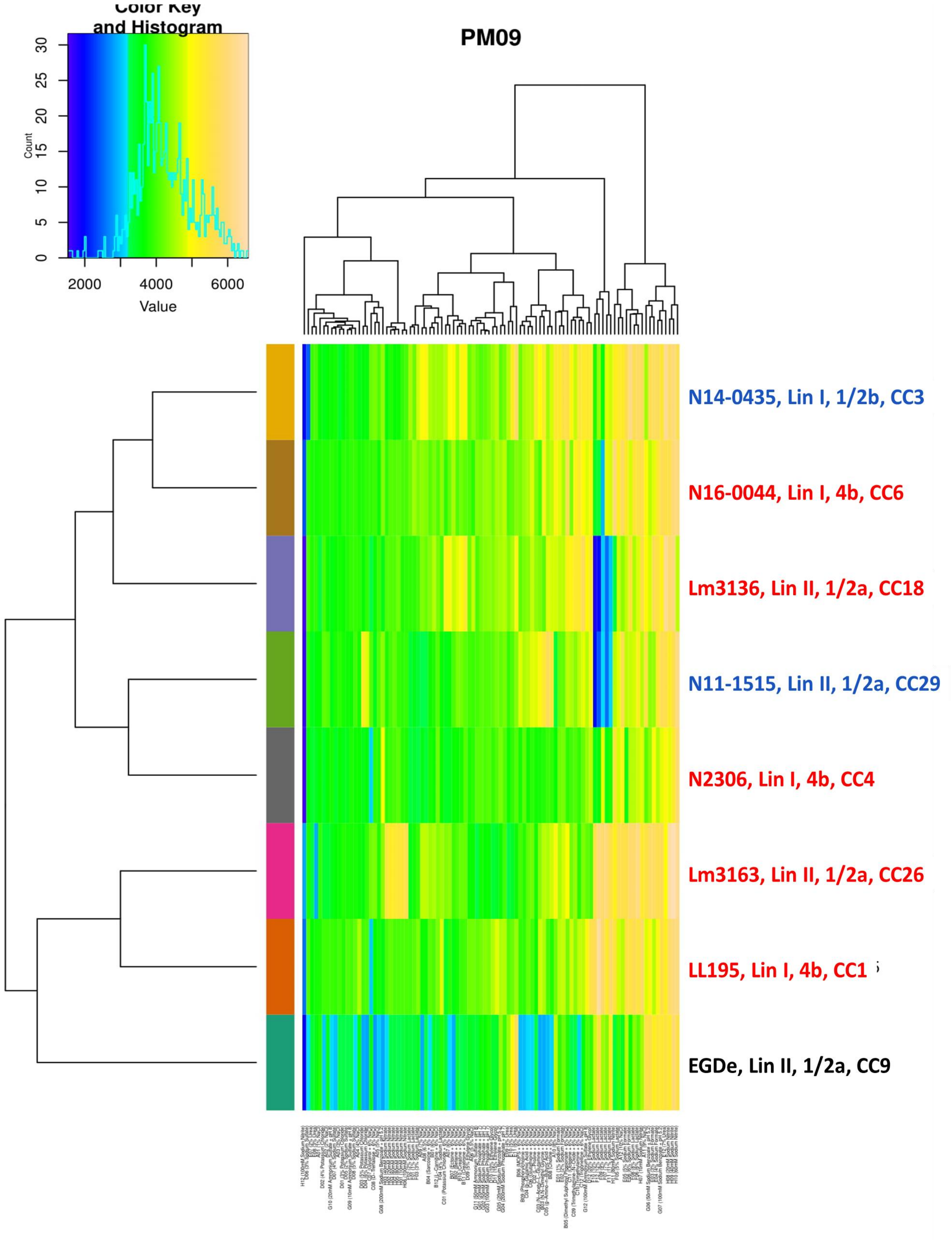

Figure S2D. Heat map showing clustering of the strains based on PM10 growth/metabolic activity results. Clinical listeriosis outbreak strains are shown in red, strains isolated from food during routine check are shown in blue and the *L. monocytogenes* EGDe reference strain is shown in black.

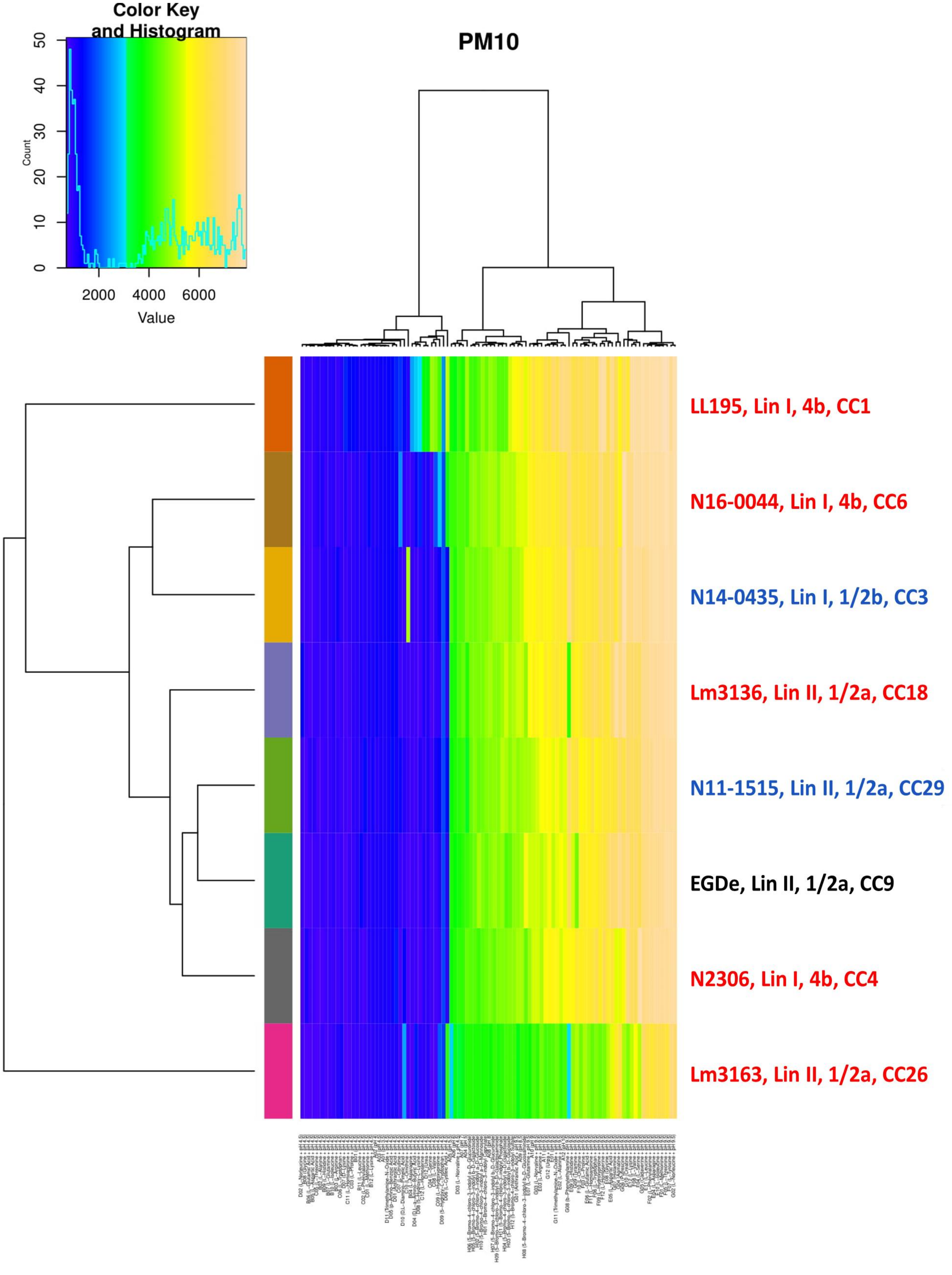

Figure S3. Respiration rate based kinetic growth assays showing that metabolic capacity among *Listeria monocytogenes* strains varied under selected food relevant osmotic stress conditions.

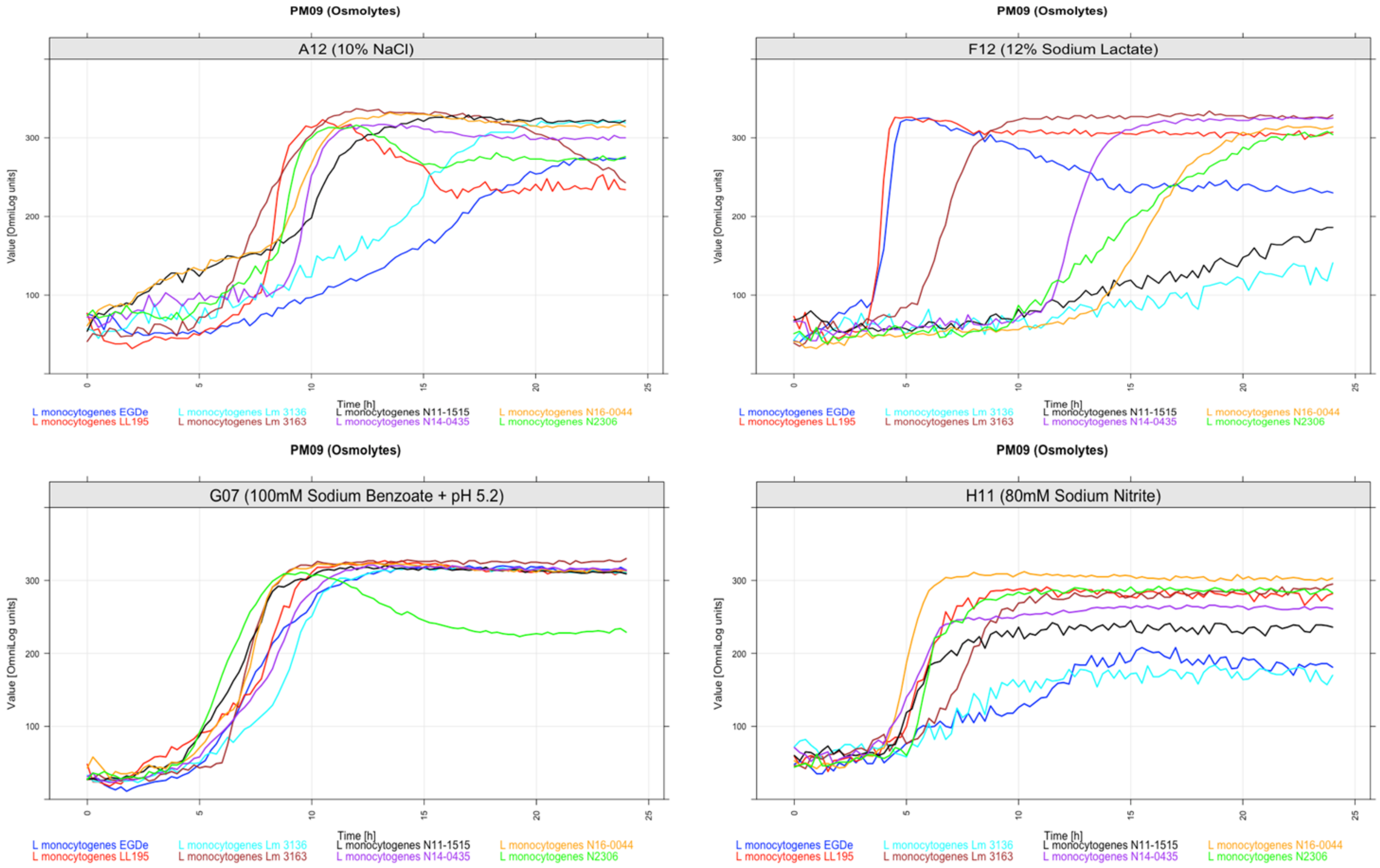

Supplement: Supplementary file 7 [file Data_Sheet_1.PDF]
